# Epidemiological change of influenza virus in hospitalized children with acute respiratory tract infection during 2014−2022 in Hubei Province, China

**DOI:** 10.1186/s12985-023-02092-1

**Published:** 2023-06-13

**Authors:** Song Yi, Wan-Xue Zhang, Yi-Guo Zhou, Xin-Rui Wang, Juan Du, Xing-Wen Hu, Qing-Bin Lu

**Affiliations:** 1 Department of Medical Genetic Center, Maternal and Child Health Hospital of Hubei Province, Wuhan, 430070 People’s Republic of China; 2 Department of Epidemiology and Biostatistics, School of Public Health, Peking University, Beijing, 100191 People’s Republic of China; 3 Department of Health Policy and Management, School of Public Health, Peking University, Beijing, 100191 People’s Republic of China; 4 Department of Laboratorial Science and Technology and Vaccine Research Center,, School of Public Health, Peking University, 38th Xueyuan Road, Haidian District, Beijing, 100191 People’s Republic of China; 5 Global Center for Infectious Disease and Policy Research and Global Health and Infectious Diseases Group, Peking University, Beijing, 100191 People’s Republic of China; 6 Department of Clinical Laboratory, Maternal and Child Health Hospital of Hubei Province, 745th Wuluo Road, Hongshan District, Wuhan, 430070 People’s Republic of China; 7 Key Laboratory of Epidemiology of Major Diseases (Peking University), Ministry of Education, Beijing, China

**Keywords:** Influenza virus, Epidemiology, The universal two-child policy, COVID−19, Hospitalized children

## Abstract

**Purpose:**

Influenza virus (IFV) causes acute respiratory tract infection (ARTI) and leads to high morbidity and mortality annually. This study explored the epidemiological change of IFV after the implementation of the universal two-child policy and evaluated the impact of coronavirus disease 2019 (COVID-19) pandemic on the detection of IFV.

**Methods:**

Hospitalized children under 18 years with ARTI were recruited from Hubei Maternal and Child Healthcare Hospital of Hubei Province from January 2014 to June 2022. The positive rates of IFV were compared among different periods by the implementation of the universal two-child policy and public health measures against COVID-19 pandemic.

**Results:**

Among 75,128 hospitalized children with ARTI, the positive rate of IFV was 1.98% (1486/75128, 95% CI 1.88–2.01). Children aged 6−17 years had the highest positive rate of IFV (166/5504, 3.02%, 95% CI 2.58−3.50). The positive rate of IFV dropped to the lowest in 2015, then increased constantly and peaked in 2019. After the universal two-child policy implementation, the positive rate of IFV among all the hospitalized children increased from 0.40% during 2014−2015 to 2.70% during 2017−2019 (RR 6.72, 95% CI 4.94−9.13, *P* < 0.001), particularly children under one year shown a violent increasing trend from 0.20 to 2.01% (RR 10.26, 95% CI 5.47−19.23, *P* < 0.001). During the initial outbreak of COVID-19, the positive rate of IFV decreased sharply compared to that before COVID-19 (0.35% vs. 3.37%, RR 0.10, 95% CI 0.04−0.28, *P* < 0.001), and then rebounded to 0.91%, lower than the level before COVID-19 (RR 0.26, 95% CI 0.20−0.36, *P* < 0.001).

**Conclusion:**

IFV epidemiological pattern has changed after the implementation of the universal two-child policy. More attention should be emphasized to comprehend the health benefits generated by COVID‐19 restrictions on IFV transmission in future.

**Supplementary Information:**

The online version contains supplementary material available at 10.1186/s12985-023-02092-1.

## Introduction

Influenza is an acute respiratory disease caused by influenza virus (IFV) infections that have led to heavy health burden [1, 2]. Circulating among the population by the infection of respiratory tract, IFV usually activates during colder periods, particularly in spring and winter in China [3, 4]. Patients with influenza mostly show self-limited upper respiratory tract infections (URTI) and some develop into lower respiratory tract infection (LRTI) with pneumonia or bronchitis [5]. Despite marvelous efforts have been made to eliminate the transmission of IFV, it is still epidemic worldwide and contributes to a billion cases and about half a million deaths annually [6]. The prevention of IFV is still one of the important global public health issues.

China had implemented the one-child policy since 1979 to alleviate population growth [7]. For the past few decades, a series of social issues such as population ageing, labor shortages were arisen, suggesting an adjustment of population policy [8]. Subsequently, starting on 1 January, 2016, the universal two-child policy was implemented by the government of China, allowing all Chinese married couples to have two children. In the era of the universal two-child policy, the number of live births increased, with a boosting newborn population in Wuhan city increasing from 100,784 in 2014 to 131,409 in 2017. This brought new challenges to the control of various infectious diseases. Changes in age structure caused by an increased number of newborns may affect the epidemiological characteristics of infectious diseases. Besides, young children infected with pathogens such as IFV can have serious symptoms resulting in hospital admissions or even death [9]. However, since the universal two-child policy was implemented, the changes of epidemiological features of IFV remain uninvestigated.

The coronavirus disease 2019 (COVID-19) is a new infectious disease that has rapidly spread worldwide through close human interactions or the contaminated secretions of the infected people, which has a similar transmission route to IFV [10]. Besides massive vaccination, nonpharmaceutical interventions (NPIs), including mask-wearing, physical distancing, hygiene promotion, and targeted restrictions on gathering and movement have been implemented and demonstrated effectiveness in limiting the spread of COVID-19 [11]. Studies have explored the impact of COVID‐19 pandemic and related NPIs on the epidemiological pattern of respiratory infections [12, 13]. Early studies reported that during the first wave of the pandemic, a nearly immediate reduction of influenza was observed, but the impact of COVID‐19 restrictions during the regular epidemic prevention and control period on IFV transmission still needs to be systematically studied [14].

This study aimed to illustrate the overall detection of IFV in Hubei Province and compare its epidemiological patterns before and after the universal two-child policy and COVID-19 pandemic. The findings might assist in the understanding of how the universal two-child policy affects the epidemiological patterns of infectious disease and may help policy makers comprehend the health benefits generated by NPIs.

## Materials and methods

### Study setting and participants

In this observational study, hospitalized children with ARTI were recruited in Hubei Maternal and Child Health Hospital, Hubei Province, China between 1 January 2014 and 30 June 2022. Hubei Maternal and Child Health Hospital undertakes the medical and health care for women and children at provincial level, dominating in the development of diagnosis and treatment management of children's respiratory diseases in Hubei Province. There are more than 3000 beds in total and nearly three million outpatients and 100,000 inpatients every year. The research was reviewed and approved by the human ethics committee of Hubei Maternal and Child Health Hospital (2022IEC052).

Hospitalized children under eighteen years with ARTI were recruited by using the case definition as follows: (1) aged under 18 years; (2) at least one of the following conditions: fever, abnormal white blood cell (WBC) differentials, leukocytosis or leukopenia; (3) at least one of the following symptoms/signs: cough, chills, sore throat, expectoration, nasal congestion, chest pain, tachypnea, and abnormal pulmonary breath sounds [15]. Pneumonia was diagnosed according to the guidelines carried by Chinese Thoracic Society [16]. The ARTI was categorized into URTI and LRTI. Hospitalized children with bronchitis and pneumonia were classified into LRTI.

### Testing and IFV detection

Within the first 24 h of admission to hospital, fresh nasopharyngeal swabs were collected from all participants. The nasopharyngeal swabs were placed in 3 mL normal saline, and transported to the laboratory and stored at − 80 °C.

The D^3^ Ultra DFA Respiratory Virus Screening & ID kit produced by DIAGNOSTIC HYBRIDS (1055 East State St., Suite 100, Athens, Ohio, USA) were used to detect IFV-A and IFV-B in the nasopharyngeal swab. After mixing on the vortex oscillator, the swab was removed. Through centrifugation at 708 g/min for 10 min, part of the supernatant was discarded, and about 100–150 μL of supernatant was left to mixed to form a turbidized cell suspension. The suspension was mixed with a straw to form a cell suspension. About 15 μL of the suspension was taken on the spot sample slide, and was first air-dried, then immersed in cold acetone solution and fixed for 10 min. Approximately 25 μL of the corresponding fluorescent antibody was added to each spot sample. Then the slides were placed in a wet box and incubated at 37 ℃ for 30 min. After removing the slides, the blocking solution was dropped and the slides were covered. A specimen was defined as positive if two or more positive cells were found in a 200-fold magnification field of view when viewed under an OLYMPUS BX53 fluorescence microscope.

### Data resource

Demographic data including gender and age, clinical data including diagnosis and IFV detection results were collected from all hospitalized children. Children were categorized into age groups of < 1 year, 1–2 years, 3–5 years and 6–17 years.

In order to analyze the effect of the universal two-child policy, the time intervals were categorized into 2014–2015 (before the universal two-child policy implementation) and 2017–2019 (one year after the universal two-child policy implementation). For the COVID-19 and related NPIs, we split the time into Stage I (the combined period during February–June of 2017–2019), Stage II (the combined period during February–June of 2020) and Stage III (the combined period during February–June of 2021 and 2022).

In this study, we defined the seasons of spring (from March to May), summer (from June to August), autumn (from September to November) and winter (from December to February next year).

### Statistical analysis

Categorical variables were summarized as frequencies and proportions. The positive rate of IFV as well as the 95% confidence interval (95% CI) were estimated by Wilson method. To evaluate the association between demographic variables (gender and age groups) and diagnosis and the positive rate of IFV, a logistic regression model was performed to calculate the adjusted odds ratio (OR) and its 95% CI.

Temporal trends of influenza and weekly positive rates of IFV were illustrated using time series plot and heatmap based on the age groups and gender in the overall, URTI and LRTI children.

To demonstrate the impact of the universal two-child policy and the COVID-19 and related NPIs on the activity of IFV, the positive rate of IFV during different periods were calculated and compared by using rate ratio (RR) as well as its 95% CI. All statistical tests were two‐sided, and a level of *P* < 0.05 was used to declare statistical significance.

Data description, graphing and analysis were conducted using R 4.2.1 (R Foundation, Vienna, Austria) and GraphPad Prism 9.3.0 (La Jolla, CA, USA).

## Results

### Characteristics of the hospitalized children and IFV detection

During 1 January 2014 to 30 June 2022, 75,128 hospitalized children under 18 years with ARTI in Hubei Maternal and Child Health Hospital were included in this study, with 44,961 (59.85%) male and 30,167 (40.15%) female (Table 1). There were 26,498 (35.27%) children under one year, 23,919 (31.84%) aged 1–2 years, 19,207 (25.57%) aged 3–5 years and 5504 (7.33%) aged 6–17 years. The diagnosis of those hospitalized children were grouped into URTI (23,922, 29.26%) and LRTI (60,049, 70.74%). Among all the children, 1486 (1.98%, 95% CI 1.88–2.01) was positive for IFV, including 954 (64.20%) for single IFV-A, 521 (35.06%) for single IFV-B and 11 (0.74%) with co-infection.

**Table 1:** The positive rate of IFV in the hospitalized children during January 2014 to June 2022

Group	No. of children (%)	Positive (%)			Positive rate (95% CI), %	OR (95% CI)	*P*
		Total	IFV-A	IFV-B			
Overall	75,128	1486 (100)	954 (64.20)	521 (35.06)	1.98 (1.88–2.01)		
*Gender*							
Male	44,961 (59.85)	882 (59.35)	580 (60.80)	295 (56.62)	1.96 (1.84–2.10)	1.01 (0.91–1.12)	0.830^a^
Female	30,167 (40.15)	604 (40.65)	374 (39.20)	226 (43.38)	2.00 (1.85–2.17)	Reference	
*Age, year*							
< 1	26,498 (35.27)	358 (24.09)	271 (28.41)	83 (15.93)	1.36 (1.22–1.50)	Reference	
1–2	23,919 (31.84)	536 (36.07)	361 (37.84)	174 (33.40)	2.24 (2.06–2.44)	1.70 (1.48–1.95)	< 0.001^b^
3–5	19,207 (25.57)	426 (28.67)	251 (26.31)	172 (33.01)	2.22 (2.01–2.44)	1.68 (1.46–1.94)	< 0.001^b^
6–17	5504 (7.33)	166 (11.17)	71 (7.44)	92 (17.66)	3.02 (2.58–3.50)	2.32 (1.92–2.80)	< 0.001^b^
*Diagnosis*							
URTI	21,983 (29.26)	420 (28.26)	248 (26.00)	170 (32.63)	1.91 (1.73–2.10)	Reference	
LRTI	53,145 (70.74)	1066 (71.74)	706 (74.00)	351 (67.37)	2.01 (1.89–2.13)	1.13 (1.01–1.27)	0.032^c^

*IFV* Influenza virus, *URTI*, Upper respiratory tract infection, *LRTI*, Lower respiratory tract infection, *CI*, Confidence interval, *OR*, Odds ratio, *P P value*

^a^Adjusted for gender and diagnosis

^b^Adjusted for age and diagnosis

^c^Adjusted for age and gender. There were 11 children mixed infection with IFV-A and IFV-B

The positive rates of IFV in different gender were similar (1.96% in male and 2.00% in female). Among different age groups, children under one year were the most but their positive rate of IFV was the lowest (1.36%, 95% CI 1.22–1.50), while children aged 6 to 17 years had the highest positive rate of IFV (3.02%, 95% CI 2.58–3.50). There were more hospitalized children with LRTI and higher positive rate of IFV than the children with URTI (70.74% vs. 29.26%, OR 1.13, 95% CI 1.01–1.27).

### Temporal distribution

**Fig. 1:**
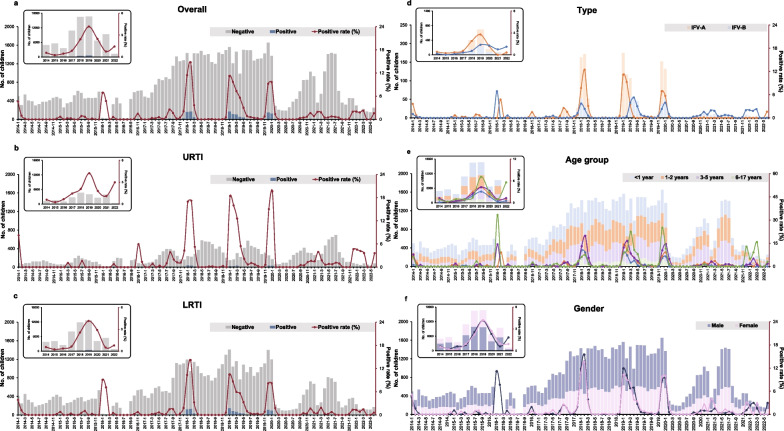
The positive rate of IFV by month in the hospitalized children during January 2014 to June 2022. **a** The positive rate of IFV among the overall children; **b** The positive rate of IFV among children with URTI; **c** The positive rate of IFV among children with LRTI; **d** The positive rate of IFV-A and IFV-B; **e** The positive rate of IFV among the overall children in different age groups; **f** The positive rate of IFV among the overall children of different gender

There were some shifts in the timing of the peak of IFV infection (Fig. 1a). For all the children and the children with URTI or LRTI, the peak of IFV infections mostly occurred during winter, and some small peaks were also observed in autumn and spring. The positive rate of IFV remained at low levels from 2014 to 2016, except for a high peak in the beginning of 2016. Since the autumn and winter in 2017, there has been a large increase in the infection of IFV. The peak of IFV infection was observed in 2019, and then a sharp decrease of IFV detections occurred. There were no IFV infections from March to August in 2020, followed by a slight increase during winter, and the IFV activity remained minimal in 2021 and 2022. Similar trends of the positive rate of IFV were observed in children with URTI and LRTI (Fig. 1b and c). For different types of IFV, a shift of the dominant type happened in 2020, with IFV-B took the predominant prevalence instead of IFV-A after 2020 (Fig. 1d). However, the epidemiological pattern of the two types of viruses shared a similar trend.

**Fig. 2:**
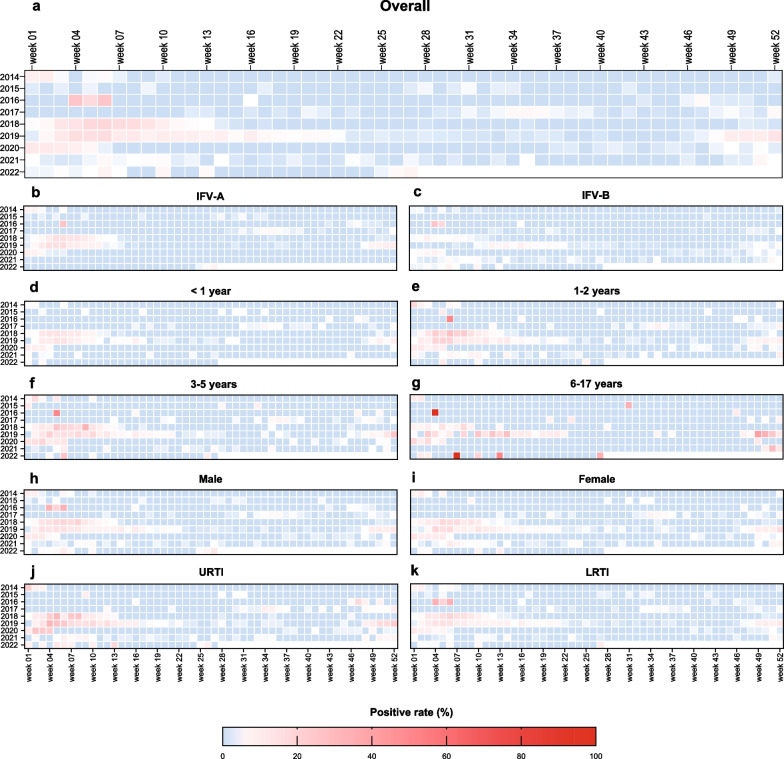
The heatmap of weekly positive rate of IFV among different diagnosis, type, gender and age groups during January 2014 to June 2022. **a** Weekly positive rate of IFV among the overall children; **b** Weekly positive rate of IFV-A among the overall children; **c** Weekly positive rate of IFV-B among the overall children; **d** Weekly positive rate of IFV among the overall children under one year; **e** Weekly positive rate of IFV among the overall children aged 1–2 years; **f** Weekly positive rate of IFV among the overall children aged 3–5 years; **g** Weekly positive rate of IFV among the overall children aged 6–17 years; **h** Weekly positive rate of IFV among the overall male children; **i** Weekly positive rate of IFV among the overall female children; **j** Weekly positive rate of IFV among children with URTI; **k** Weekly positive rate of IFV among children with LRTI

Weekly positive rate of IFV revealed that there were some differences in the prevalence and the timing of the influenza peak from year to year (Fig. 2). In general, influenza mostly activated in the few weeks at the beginning and end of the years. Longer time periods of IFV detection were observed in 2018 and 2019, with a constant positivity of IFV until week 13 and week 22, respectively.

Similar temporal epidemiological patterns were found among different age groups and gender (Fig. 1e and c, Additional files 1: Fig. S1 and 2: Fig. S2). The positive rate of IFV shown no difference between male and female. The distribution of the prevalence of influenza was similar in different age groups. The positive rate was highest in the group of 6–17 years, followed by the groups of 1–2 years, 3–5 years and < 1 year.

### Impact of the universal two-child policy on IFV detection

The positive rate of IFV in overall children was 0.40% during 2014 to 2015, while there was a huge increase to 2.70% during 2017**–**2019 after the policy implementation (RR 6.72, 95% CI 4.94–9.13, *P* < 0.001) (Table 2). The same trend was observed in children with URTI (from 0.41% to 2.67%, RR 6.54, 95% CI 3.59–11.91, *P* < 0.001) and LRTI (from 0.40% to 2.71%, RR 6.78, 95% CI 4.74–9.69, *P* < 0.001).

**Table 2:** The positive rate of IFV in Hubei Province stratified by the universal two–child policy implementation

Group	2014–2015			2017–2019			RR (95% CI)	*P*
	No. of children	Positive	Positive rate (95% CI), %	No. of children	Positive	Positive rate (95% CI), %		
Overall	10,446	42	0.40 (0.30–0.54)	43,916	1186	2.70 (2.55–2.86)	6.72 (4.94–9.13)	< 0.001
*Gender*								
Male	6438	27	0.42 (0.29–0.61)	26,183	712	2.72 (2.53–2.92)	6.48 (4.42–9.51)	< 0.001
Female	4008	15	0.37 (0.23–0.62)	17,733	474	2.67 (2.45–2.92)	7.14 (4.28–11.93)	< 0.001
*Age, year*								
< 1	5105	10	0.20 (0.11–0.36)	14,977	301	2.01 (1.80–2.25)	10.26 (5.47–19.24)	< 0.001
1–2	2984	16	0.54 (0.33–0.87)	14,248	429	3.01 (2.74–3.30)	5.62 (3.41–9.23)	< 0.001
3–5	1850	13	0.70 (0.41–1.20)	11,224	321	2.86 (2.57–3.18)	4.07 (2.34–7.07)	< 0.001
6–17	507	3	0.59 (0.20–1.73)	3467	135	3.89 (3.30–4.59)	6.58 (2.10–20.58)	< 0.001
URTI	2691	11	0.41 (0.23–0.73)	11,340	303	2.67 (2.39–2.99)	6.54 (3.59–11.91)	< 0.001
*Gender*								
Male	1559	7	0.45 (0.22–0.92)	6682	175	2.62 (2.26–3.03)	5.83 (2.75–12.39)	< 0.001
Female	1132	4	0.35 (0.14–0.90)	4658	128	2.75 (2.32–3.26)	7.78 (2.88–20.99)	< 0.001
*Age, year*								
< 1	1001	5	0.50 (0.21–1.16)	2713	57	2.10 (1.63–2.71)	4.21 (1.69–10.46)	< 0.001
1–2	956	2	0.21 (0.06–0.76)	4164	111	2.67 (2.22–3.20)	12.74 (3.15–51.49)	< 0.001
3–5	536	2	0.37 (0.10–1.35)	3282	88	2.68 (2.18–3.29)	7.19 (1.77–29.10)	< 0.001
6–17	198	2	1.01 (0.28–3.61)	1181	47	3.98 (3.01–5.25)	3.94 (0.96–16.09)	< 0.001
LRTI	7755	31	0.40 (0.28–0.57)	32,576	883	2.71 (2.54–2.89)	6.78 (4.74–9.69)	< 0.001
*Gender*								
Male	4879	20	0.41 (0.27–0.63)	19,501	537	2.75 (2.53–2.99)	6.72 (4.30–10.49)	< 0.001
Female	2876	11	0.38 (0.21–0.68)	13,075	346	2.65 (2.38–2.94)	6.92 (3.80–12.59)	< 0.001
*Age, year*								
< 1	4104	5	0.12 (0.05–0.28)	12,264	244	1.99 (1.76–2.25)	16.33 (6.74–39.56)	< 0.001
1–2	2028	14	0.69 (0.41–1.16)	10,084	318	3.15 (2.83–3.51)	4.57 (2.68–7.78)	< 0.001
3–5	1314	11	0.84 (0.47–1.49)	7942	233	2.93 (2.58–3.33)	3.50 (1.92–6.40)	< 0.001
6–17	309	1	0.32 (0.06–1.81)	2286	88	3.85 (3.14–4.72)	11.90 (1.66–85.08)	< 0.001

*IFV* influenza virus, *URTI* upper respiratory tract infection, *LRTI* lower respiratory tract infection, *CI* confidence interval, *RR* rate ratio, *P P value*

Compared to the period before the policy implemented, the effect values of RR were higher in children under one year in the overall and LRTI group, from 0.20% to 2.01% (RR 10.26, 95% CI 5.47–19.24, *P* < 0.001), 0.12% to 1.99% (RR 16.33, 95% CI 6.74–39.56, *P* < 0.001), respectively. For the children with URTI, children aged 1–2 years shown the biggest change of IFV positive rate (RR 12.74, 95% CI 3.15–51.49, *P* < 0.001).

### Impact of the COVID-19 pandemic and NPIs on IFV detection

**Fig. 3:**
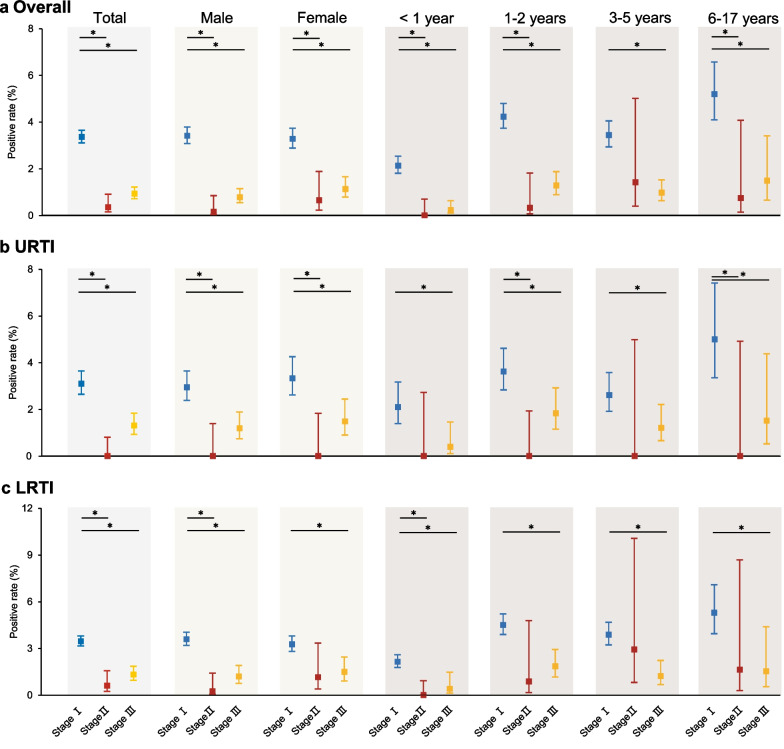
Dynamics of the positive rate of IFV before and after COVID-19 in Hubei Province from 2014 to 2021. **a** The positive rate of IFV among the overall children during three stages; **b** The positive rate of IFV among children with URTI during three stages; **c** The positive rate of IFV among children with LRTI during three stages. The blue line presents the positive rate of IFV during Stage I, the combined period during February–June of 2017–2019. The red line presents the positive rate of IFV during Stage II, the combined period during February–June of 2020. The yellow line presents the positive rate of IFV during State III, the combined period during February–June of 2021 and 2022. “*” was represented *P* < 0.05. COVID-19, coronavirus disease 2019

Hospitalized children were divided into three stages according to the outbreak of COVID-19. For all the children, the positive rate of IFV during Stage I (3.37%, 95% CI 3.11–3.65) was significantly higher compared to that during Stage II (0.35%, 95% CI 0.14–0.90, RR 0.10, 95% CI 0.04–0.28, *P *< 0.001) and Stage III (0.91%, 95% CI 0.69–1.19, RR 0.26, 95% CI 0.20–0.36, *P *< 0.001) (Fig. 3a and Additional file 3: Table S1). Among all the children, different gender and age groups were further analyzed. Except for children aged 3–5 years, children with URTI under one year, female children and children aged 1–2 years, 3–5 years and 6–17 years with LRTI, the positive rate of IFV in stage II was significantly lower than that in stage I (*P *< 0.05), respectively. During the regular epidemic prevention and control period in 2021 and 2022 after the outbreak of COVID-19, we observed a slight increase in the frequency of IFV detection, but the positive rate was still significantly lower than that in the same period during 2017–2019.

## Discussion

Our results showed the changing pattern of IFV in hospitalized children with ARTI during 2014–2022 varied year by year. The overall positive rate of IFV increased after the implementation of the universal two-child policy. The incidence pattern of IFV had significant reductions during the time of COVID‐19 outbreak and related NPIs implemented, but those measures did not continuously sustain IFV infections at low levels. The rebound of IFV infections was observed during the regular epidemic prevention and control period in 2021 and 2022.

Consistent with prior studies, the positive rate of IFV in children varied by gender and age, which may be related to the different vaccination rate, immune system ability and behavior patterns [17]. This study showed the positive rate of IFV in patients with LRTI was higher than that in patients with URTI, reminding that IFV is an important pathogen associated with LRTI [18]. Strengthening surveillance for IFV is critical for preventing illness and reducing hospitalization [19].

The influenza seasonality observed in our study was similar to other regions such as Chongqing city, which located at the same latitude as Hubei Province [20]. The main peak occurred during winter, since influenza activity frequently followed low seasonal temperatures [21]. Besides, children’s summer vacations reduce the frequency of them gathering and interacting, preventing the IFV from being transmitted among children. The impact of other climate conditions such as humidity, precipitation and sunshine on the IFV activity were also found in some studies, which may explain the difference of the specific epidemic patterns every year [22, 23]. During the influenza seasons, the IFV-A co-circulated with the IFV-B and there were some co-infections occurred. Same phenomenon has been described in other studies conducted in the same area [24, 25]. It should be noted that after the outbreak of COVID-19, IFV-B is predominant in hospitalized children instead of IFV-A. This transition may be related to that children infected with IFV-A have a higher frequency of symptoms, leading to more medical attention and early identification of children at higher risk during the COVID-19 pandemic, which may reduce the proportion of hospitalization [26]. Besides, the COVID-19 may have an impact on the types of IFV, which needs to be further investigated.

Implemented on 1 January 2016, the universal two child policy has been associated with a rise in births in China [27]. In 2017–2019, the prevalence of IFV in hospitalized children increased, and children under one year had demonstrated a rapid boost in the positive rate of IFV. Newborns increased the population density in family, causing more exposure and transmission within households especially in the infants who lacks of protective antibodies [28]. What’s more, evidence provided by previous studies suggested that there were more multiparous mothers and mother aged 35 years and over on the scale, increasing the risk of birth outcomes such as preterm birth and birth defects [27, 29–31]. With unmature immune system, the burden of newborn infectious disease has always been considered as the highest across the entire human life span [32]. We evaluated the impact of this policy on the epidemiology of influenza with a large sample size. Our findings suggest that with the implementation of the universal two-child policy, influenza prevention and control targeted to the children especially newborns should be strengthened and developed in facing the assault of increasing susceptible population.

In the early stage of COVID-19, the spread of influenza reduced, implying that rigorous implementation of NPIs was likely to benefit the control of influenza [33, 34]. Studies conducted in other regions and countries have shown similar results in the epidemiology of influenza and other respiratory viral diseases [13, 35, 36]. In Western Australia, the COVID-19 related NPIs also reduced the circulation of respiratory syncytial virus, altering its seasonality [37]. At the beginning of COVID-19 outbreak, Wuhan city was closed and traffic restricted under the strong control of the government. Almost all people were quarantined at home, minimizing the transmission of the infection to others [14]. A series of measures were taken to limit the possibility of IFV transmission.

After success in early elimination local transmission of severe acute respiratory syndrome coronavirus-2 (SAR-CoV-2), the resumption of work and life was advanced, followed by a resurgence of influenza infections [38]. The rebound of influenza was likely attributed to the accumulation in population susceptibility coupled with relaxed NPIs. The decline in child vaccination coverage during COVID-19 outbreak increased the risk of infection [39]. Besides, the threshold for respiratory virus testing in the context of the regular epidemic prevention and control for COVID-19 was lowered, facilitating more patients get tested and virus get detected [12].

However, the epidemic level of influenza has not returned to the level before COVID-19. This phenomenon reveals that COVID‐19 outbreak and related NPIs has altered influenza epidemiology and seasonality. Daily protective measures such as mask wearing have interrupted a significant portion of the transmission of IFV [40]. However, large number of people wear mask for long periods of time may increase population susceptibility to pathogens, which may lead to a huge bounce of infectious disease. Influenza vaccine has demonstrated effectiveness in the prevention of influenza and reducing severe disease [41]. Promoting the influenza vaccination to increase vaccination coverage in the population is still vital to prevent the epidemic of influenza.

There were several limitations in this research. Firstly, this study was a retrospective analysis based on historical data, so there are some unmeasured biases and the causal inferences cannot be concluded. Secondly, only outpatient under 18 years in a single center were included in our study, lacking more data from the population to illustrate the whole situation. Due to the impact of COVID-19 outbreak, the hospitalization and the isolation of the viruses may be delayed and suspended, possibly leading to biased results.

## Conclusion

IFV has been activating continuously in Hubei Province. The universal two-child policy increased the possibility of IFV transmission especially in newborns. Influenza activity among children declined sharply at the beginning of COVID-19 outbreak and rebounded during the regular epidemic prevention and control period in 2021 and 2022. NPIs has altered influenza epidemiology. More attention should be emphasized to comprehend the health benefits generated by COVID‐19 restrictions on IFV transmission in future.

## Supplementary Information

**Additional file 1: Figure S1.** The positive rate of IFV among children with URTI and LRTI in different gender and age groups during January 2014 to June 2022.The positive rate of IFV−A and IFV−B among children with URTI;The positive rate of IFV among children with URTI in different age groups;The positive rate of IFV among children with URTI in different gender;The positive rate of IFV−A and IFV−B among children with LRTI;The positive rate of IFV among children with LRTI in different age groups;The positive rate of IFV among children with LRTI in different gender.

**Additional file 2: Figure S2.** The heatmap of weekly positive rate of IFV among children with URTI and LRTI in different diagnosis, gender and age groups during January 2014 to June 2022. Weekly positive rate of IFV−A and IFV−B among children with URTI;Weekly positive rate of IFV among children with URTI in different gender;Weekly positive rate of IFV among children with URTI in different age groups;Weekly positive rate of IFV−A and IFV−B among children with LRTI;Weekly positive rate of IFV among children with LRTI in different gender;Weekly positive rate of IFV among children with LRTI in different age groups.

**Additional file 3: Table S1.** The positive rate of IFV in Hubei Province from 2017-2022, stratified by COVID-19 outbreak.

## Data Availability

The datasets used and/or analyzed during the current study are available from the corresponding author on reasonable request.
