# Supplementary figures and images for: Epidemiological change of influenza virus in hospitalized children with acute respiratory tract infection during 2014−2022 in Hubei Province, China
Source: Virol J. 2023 Jun 13;20:122. doi: 10.1186/s12985-023-02092-1 (PMC10262143; doi:10.1186/s12985-023-02092-1)

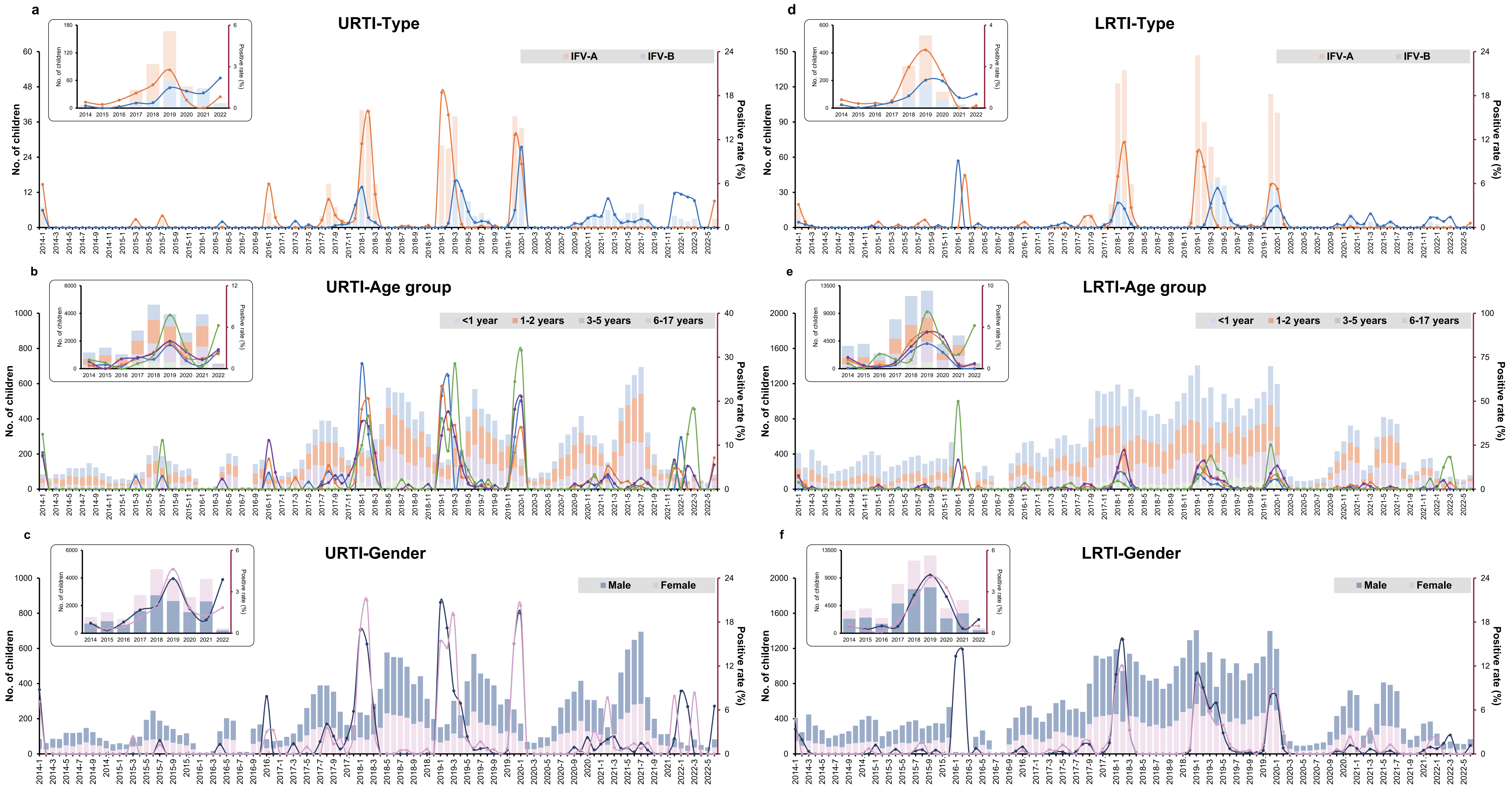

Supplement: Supplementary file 1 — Additional file 1: Figure S1. The positive rate of IFV among children with URTI and LRTI in different gender and age groups during January 2014 to June 2022.The positive rate of IFV−A and IFV−B among children with URTI;The positive rate of IFV among children with URTI in different age groups;The positive rate of IFV among children with URTI in different gender;The positive rate of IFV−A and IFV−B among children with LRTI;The positive rate of IFV among children with LRTI in different age groups;The positive rate of IFV among children with LRTI in different gender. [file 12985_2023_2092_MOESM1_ESM.pdf]

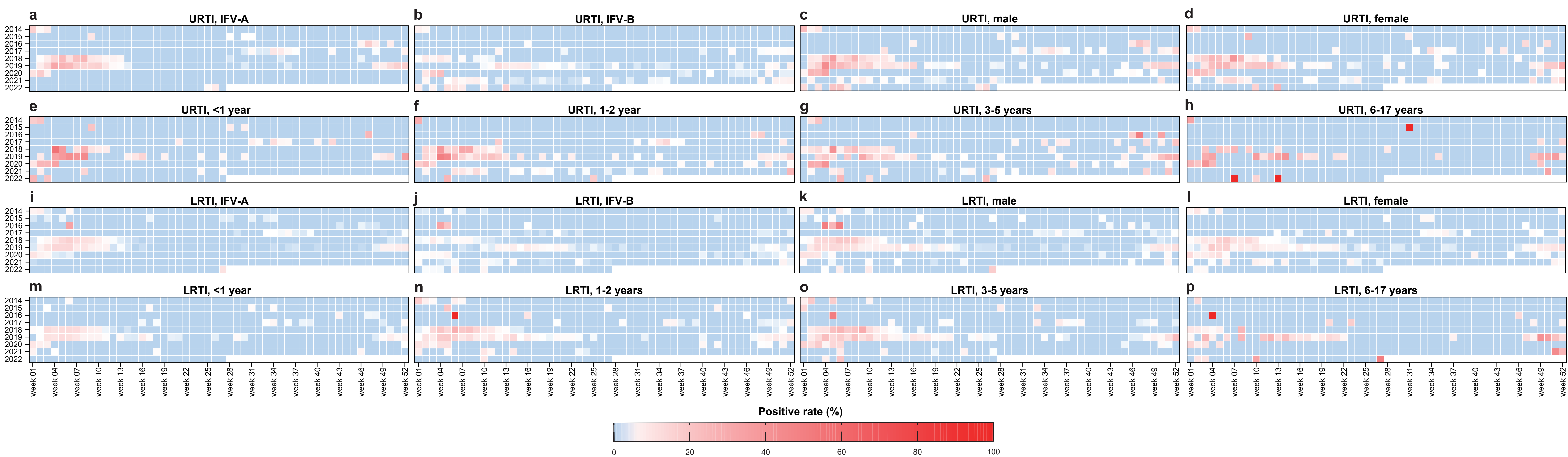

Supplement: Supplementary file 2 — Additional file 2: Figure S2. The heatmap of weekly positive rate of IFV among children with URTI and LRTI in different diagnosis, gender and age groups during January 2014 to June 2022. Weekly positive rate of IFV−A and IFV−B among children with URTI;Weekly positive rate of IFV among children with URTI in different gender;Weekly positive rate of IFV among children with URTI in different age groups;Weekly positive rate of IFV−A and IFV−B among children with LRTI;Weekly positive rate of IFV among children with LRTI in different gender;Weekly positive rate of IFV among children with LRTI in different age groups. [file 12985_2023_2092_MOESM2_ESM.pdf]
