# Supplementary material for: Epidemiological change of influenza virus in hospitalized children with acute respiratory tract infection during 2014−2022 in Hubei Province, China
Source: Virol J. 2023 Jun 13;20:122. doi: 10.1186/s12985-023-02092-1 (PMC10262143; doi:10.1186/s12985-023-02092-1)
Supplement: Supplementary file 3 — Additional file 3: Table S1. The positive rate of IFV in Hubei Province from 2017-2022, stratified by COVID-19 outbreak. [file 12985_2023_2092_MOESM3_ESM.docx]

Supplemental Table 1. Positive rate of IFV in Hubei Province from 2017-2022, stratified by COVID-19 outbreak

| Group | No. of children | Positive | Positive rate (95% CI), % | RR (95% CI) | *P* |
| --- | --- | --- | --- | --- | --- |
| Overall |  |  |  |  |  |
| Stage Ⅰ | 17010 | 573 | 3.37 (3.11-3.65) | Reference |  |
| Stage Ⅱ | 1135 | 4 | 0.35 (0.14-0.90) | 0.10 (0.04-0.28) | < 0.001 |
| Stage Ⅲ | 5744 | 52 | 0.91 (0.69-1.19) | 0.26 (0.20-0.36) | < 0.001 |
| Gender |  |  |  |  |  |
| Male |  |  |  |  |  |
| Stage Ⅰ | 10143 | 347 | 3.42 (3.08-3.79) | Reference |  |
| Stage Ⅱ | 670 | 1 | 0.15 (0.03-0.84) | 0.04 (0.01-0.31) | < 0.001 |
| Stage Ⅲ | 3407 | 26 | 0.76 (0.52-1.12) | 0.22 (0.15-0.33) | < 0.001 |
| Female |  |  |  |  |  |
| Stage Ⅰ | 6867 | 226 | 3.29 (2.89-3.74) | Reference |  |
| Stage Ⅱ | 465 | 3 | 0.65 (0.22-1.88) | 0.19 (0.06-0.61) | 0.001 |
| Stage Ⅲ | 2337 | 26 | 1.11 (0.76-1.63) | 0.33 (0.23-0.51) | < 0.001 |
| Age, year |  |  |  |  |  |
| <1 |  |  |  |  |  |
| Stage Ⅰ | 6062 | 130 | 2.14 (1.81-2.54) | Reference |  |
| Stage Ⅱ | 550 | 0 | 0.00 (0.00-0.69) | 0 | < 0.001 |
| Stage Ⅲ | 1456 | 3 | 0.21 (0.07-0.60) | 0.09 (0.03-0.30) | < 0.001 |
| 1-2 |  |  |  |  |  |
| Stage Ⅰ | 5497 | 233 | 4.24 (3.74-4.80) | Reference |  |
| Stage Ⅱ | 309 | 1 | 0.32 (0.06-1.81) | 0.07 (0.01-0.54) | < 0.001 |
| Stage Ⅲ | 2056 | 26 | 1.26 (0.86-1.85) | 0.29 (0.20-0.45) | < 0.001 |
| 3-5 |  |  |  |  |  |
| Stage Ⅰ | 4200 | 145 | 3.45 (2.94-4.05) | Reference |  |
| Stage Ⅱ | 141 | 2 | 1.42 (0.39-5.02) | 0.40 (0.10-1.64) | 0.189 |
| Stage Ⅲ | 1891 | 18 | 0.95 (0.60-1.50) | 0.27 (0.17-0.45) | < 0.001 |
| 6-17 |  |  |  |  |  |
| Stage Ⅰ | 1251 | 65 | 5.20 (4.10-6.57) | Reference |  |
| Stage Ⅱ | 135 | 1 | 0.74 (0.13-4.08) | 0.14 (0.02-1.02) | 0.021 |
| Stage Ⅲ | 341 | 5 | 1.47 (0.63-3.39) | 0.27 (0.11-0.70) | 0.003 |
| URTI |  |  |  |  |  |
| Overall |  |  |  |  |  |
| Stage Ⅰ | 4659 | 145 | 3.11 (2.65-3.65) | Reference |  |
| Stage Ⅱ | 479 | 0 | 0.00 (0.00-0.80) | 0 | < 0.001 |
| Stage Ⅲ | 2442 | 32 | 1.31 (0.93-1.84) | 0.41 (0.29-0.62) | < 0.001 |
| Gender |  |  |  |  |  |
| Male |  |  |  |  |  |
| Stage Ⅰ | 2775 | 82 | 2.95 (2.39-3.65) | Reference |  |
| Stage Ⅱ | 273 | 0 | 0.00 (0.00-1.39) | 0 | 0.004 |
| Stage Ⅲ | 1434 | 17 | 1.19 (0.74-1.89) | 0.39 (0.24-0.67) | < 0.001 |
| Female |  |  |  |  |  |
| Stage Ⅰ | 1884 | 63 | 3.34 (2.62-4.26) | Reference |  |
| Stage Ⅱ | 206 | 0 | 0.00 (0.00-1.83) | 0 | 0.008 |
| Stage Ⅲ | 1008 | 15 | 1.49 (0.90-2.44) | 0.44 (0.25-0.78) | 0.003 |
| Age, year |  |  |  |  |  |
| <1 |  |  |  |  |  |
| Stage Ⅰ | 1041 | 22 | 2.11 (1.4-3.18) | Reference |  |
| Stage Ⅱ | 137 | 0 | 0.00 (0.00-2.73) | 0 | 0.086 |
| Stage Ⅲ | 494 | 2 | 0.40 (0.11-1.46) | 0.19 (0.05-0.81) | 0.012 |
| 1-2 |  |  |  |  |  |
| Stage Ⅰ | 1709 | 62 | 3.63 (2.84-4.62) | Reference |  |
| Stage Ⅱ | 195 | 0 | 0.00 (0.00-1.93) | 0 | 0.007 |
| Stage Ⅲ | 925 | 17 | 1.84 (1.15-2.92) | 0.50 (0.30-0.86) | 0.010 |
| 3-5 |  |  |  |  |  |
| Stage Ⅰ | 1450 | 38 | 2.62 (1.92-3.58) | Reference |  |
| Stage Ⅱ | 73 | 0 | 0.00 (0.00-5.00) | 0 | 0.161 |
| Stage Ⅲ | 826 | 10 | 1.21 (0.66-2.21) | 0.46 (0.23-0.92) | 0.024 |
| 6-17 |  |  |  |  |  |
| Stage Ⅰ | 459 | 23 | 5.01 (3.36-7.41) | Reference |  |
| Stage Ⅱ | 74 | 0 | 0.00 (0.00-4.93) | 0 | 0.049 |
| Stage Ⅲ | 197 | 3 | 1.52 (0.52-4.38) | 0.29 (0.09-1.00) | 0.036 |
| LRTI |  |  |  |  |  |
| Overall |  |  |  |  |  |
| Stage Ⅰ | 12351 | 428 | 3.47 (3.16-3.80) | Reference |  |
| Stage Ⅱ | 656 | 4 | 0.61 (0.24-1.56) | 0.17 (0.07-0.47) | < 0.001 |
| Stage Ⅲ | 3302 | 20 | 0.61 (0.39-0.93) | 0.17 (0.11-0.27) | < 0.001 |
| Gender |  |  |  |  |  |
| Male |  |  |  |  |  |
| Stage Ⅰ | 7368 | 265 | 3.60 (3.20-4.05) | Reference |  |
| Stage Ⅱ | 397 | 1 | 0.25 (0.04-1.41) | 0.07 (0.01-0.50) | < 0.001 |
| Stage Ⅲ | 1973 | 9 | 0.46 (0.24-0.86) | 0.12 (0.07-0.25) | < 0.001 |
| Female |  |  |  |  |  |
| Stage Ⅰ | 4983 | 163 | 3.27 (2.81-3.80) | Reference |  |
| Stage Ⅱ | 259 | 3 | 1.16 (0.39-3.35) | 0.35 (0.11-1.10) | 0.058 |
| Stage Ⅲ | 1329 | 11 | 0.83 (0.46-1.48) | 0.25 (0.14-0.46) | < 0.001 |
| Age, year |  |  |  |  |  |
| <1 |  |  |  |  |  |
| Stage Ⅰ | 5021 | 108 | 2.15 (1.78-2.59) | Reference |  |
| Stage Ⅱ | 413 | 0 | 0.00 (0.00-0.92) | 0 | 0.003 |
| Stage Ⅲ | 962 | 1 | 0.10 (0.02-0.59) | 0.05 (0.01-0.35) | < 0.001 |
| 1-2 |  |  |  |  |  |
| Stage Ⅰ | 3788 | 171 | 4.51 (3.90-5.22) | Reference |  |
| Stage Ⅱ | 114 | 1 | 0.88 (0.16-4.80) | 0.19 (0.03-1.38) | 0.062 |
| Stage Ⅲ | 1131 | 9 | 0.80 (0.42-1.51) | 0.17 (0.09-0.34) | < 0.001 |
| 3-5 |  |  |  |  |  |
| Stage Ⅰ | 2750 | 107 | 3.89 (3.23-4.68) | Reference |  |
| Stage Ⅱ | 68 | 2 | 2.94 (0.81-10.10) | 0.75 (0.19-3.00) | 0.688 |
| Stage Ⅲ | 1065 | 8 | 0.75 (0.38-1.48) | 0.19 (0.09-0.39) | < 0.001 |
| 6-17 |  |  |  |  |  |
| Stage Ⅰ | 792 | 42 | 5.30 (3.95-7.09) | Reference |  |
| Stage Ⅱ | 61 | 1 | 1.64 (0.29-8.72) | 0.30 (0.04-2.21) | 0.208 |
| Stage Ⅲ | 144 | 2 | 1.39 (0.38-4.92) | 0.25 (0.06-1.07) | 0.040 |

Note: IFV, influenza virus; URTI, upper respiratory tract infection; LRTI, lower respiratory tract infection; CI, confidence interval; RR, rate ratio; Stage I, the combined period during February− June of 2017-2019; Stage II, the combined period during February− June of 2020; State III, the combined period during February− June of 2021 and 2022.
